# Multiomics Characterization of GCSH + Macrophages Reveals Therapeutic Vulnerabilities and Immune–Metabolic Crosstalk in Triple‐Negative Breast Cancer

**DOI:** 10.1155/humu/7893655

**Published:** 2026-05-06

**Authors:** Jiahao Ge, Ting Chen, Yuanyuan Ma, Shuzhen Wei

**Affiliations:** ^1^ Department of Hepatobiliary and Pancreatic Surgery, Jinhua Hospital Affiliated to Zhejiang University, Jinhua, Zhejiang, China; ^2^ Department of Oncology, The Second People’s Hospital of Huai’an, The Affiliated Huai’an Hospital of Xuzhou Medical University The Second People’s Hospital of Huai’an, Jiangsu, China, xzmc.edu.cn; ^3^ Department of Radiation Oncology, The Second People’s Hospital of Huai’an, The Affiliated Huai’an Hospital of Xuzhou Medical University, Huai’an, Jiangsu, China, xzmc.edu.cn; ^4^ Department of Oncology, Huai’an 82 Hospital, China RongTong Medical Healthcare Group Co.Ltd, Huai’an, Jiangsu, China

**Keywords:** cuproptosis, GCSH, multiomics, targeted therapy, triple-negative breast cancer

## Abstract

**Background:**

Tumor‐associated macrophages (TAMs) are key regulators of immune homeostasis within the tumor microenvironment (TME) and play critical roles in malignant progression. However, the molecular mechanisms linking macrophage metabolic remodeling to immune regulation remain incompletely understood. Glycine cleavage system H protein (GCSH), a core regulator of copper‐dependent cell death, has been implicated in metabolic regulation in triple‐negative breast cancer (TNBC), suggesting a potential role in macrophage‐mediated TME remodeling.

**Methods:**

We integrated single‐cell RNA sequencing and spatial transcriptomic data from TNBC tissues to systematically characterize macrophage subpopulations with high GCSH expression. Pseudotime trajectory analysis, cuproptosis‐related scoring, cell–cell communication inference, metabolic pathway enrichment, and spatial localization analyses were performed to delineate their functional heterogeneity and microenvironmental context. In addition, mutation profiling, immunogenomic analysis, drug sensitivity prediction, and in vitro and in vivo functional experiments were conducted to comprehensively evaluate the biological and therapeutic relevance of GCSH.

**Results:**

GCSH expression was predominantly enriched in macrophages, particularly in early activated subsets, and was associated with enhanced amino acid and lipid metabolic activity. GCSH + macrophages exhibited extensive interactions with T cells via pathways such as MIF–CD74–CXCR4 and LGALS9–CD45, contributing to an immunosuppressive, tumor‐promoting microenvironment. Spatial analysis revealed their preferential localization at the tumor core–stroma interface. Notably, GCSH missense mutations were associated with increased M1 macrophage infiltration and enrichment of immune and inflammatory pathways. Clinically, high GCSH expression correlated with poor survival, genomic instability, and chemotherapy resistance. Functional experiments demonstrated that GCSH silencing suppressed tumor cell proliferation, migration, and clonogenicity, induced apoptosis, enhanced proinflammatory cytokine secretion, and significantly inhibited tumor growth in vivo.

**Conclusion:**

GCSH acts as a central molecular link between macrophage metabolic reprogramming, immune suppression, and TNBC progression, highlighting its potential as both a prognostic biomarker and therapeutic target.

## 1. Introduction

In the context of tumor immunology, tumor‐associated macrophages (TAMs), as the immune cell population with the highest infiltration density in the TME, play a core regulatory role in tumor initiation, progression, and immune evasion mechanisms [1]. Although TAMs exhibit significant phenotypic plasticity under microenvironmental signal induction, emerging research evidence suggests that their functional heterogeneity is not limited by traditional binary polarization models, but rather deeply coupled with their metabolic reprogramming processes [2]. Based on this, elucidating the metabolic regulatory factors that drive the evolution and spatial distribution of TAMs has become a current focus of research. However, the specific contribution of metabolism‐related genes in TAM immune remodeling and spatial heterogeneity is still unclear. In particular, there is still a lack of systematic research on whether the glycine cleavage system H protein (GCSH) mediates the immune metabolic axis regulation of TAMs and thereby affects the spatial diversity within tumors, which constitutes the core scientific question of this project.

Recently, copper‐mediated cell death (cuproptosis) driven by copper ion accumulation and protein thiolation modification has been shown to be closely related to tumor metabolism and immune regulation as a novel regulatory cell death mode [3] [4]. Unlike apoptosis or ferroptosis, cuproptosis directly targets mitochondrial metabolism by inducing protein toxicity stress, indicating a unique biological association between metabolic remodeling and immune cell function [5]. Given that macrophages are one of the most metabolically active immune cells in TME, their polarization state and effector function are highly dependent on mitochondrial homeostasis and metabolic adaptability. Based on this premise, cuproptosis‐related genes may exert specific lineage effects in macrophages by regulating mitochondrial homeostasis and metabolic adaptation. In addition, although the potential value of cuproptosis in regulating the tumor immune landscape has been preliminarily demonstrated [6], its specific mechanism of action in macrophage subpopulations is still in the exploratory stage [7].

As a core enzyme involved in the decarboxylation of glycine in the mitochondrial matrix, GCSH has recently been confirmed as a pivotal component of the Cuproptosis signaling pathway [3]. Although the dysregulation of GCSH expression has been confirmed to be closely related to mitochondrial metabolic disorders and redox homeostasis imbalances in various malignant tumors, its biological functions in the field of immunology have not been fully elucidated [8] [9] [10] [11]. Especially, whether GCSH is involved in mediating the functional heterogeneity of TAMs and how it intervenes in the tumor immune interaction interface remains an urgent academic topic to be addressed.

Based on this, this study systematically explored the expression pattern and functional logic of GCSH in tumor‐infiltrating macrophages using the integrated analysis technology of single‐cell transcriptome sequencing (scRNA seq) and spatial transcriptomics. Through bioinformatics methods, we identified a subpopulation of macrophages characterized by high expression of GCSH. The subsequent pseudotime analysis revealed that the expression level of GCSH reached its peak during the “early transcriptional‐like” stage of macrophage evolution in the TME. In addition, cell communication analysis showed that the GCSH high cell subset exhibited significant “strong signal reception, weak signal output” characteristics in the macrophage migration inhibitory factor (MIF) signaling pathway [12], suggesting that it may participate in mediating immune suppression processes through metabolic dialogue. Based on this inference, the signal transduction pattern of this subgroup in the microenvironment has the potential to contribute to immune escape. Furthermore, we have finely characterized the interaction between GCSH high macrophages and T cells, clarifying the specific cellular participants and their core signaling axes in the MIF regulatory network.

By integrating single‐cell transcriptome and spatial transcriptome (ST) data, this study further characterized the metabolic activity and spatial distribution characteristics of GCSH high macrophages in the tumor core area, invasion edge, and adjacent normal tissues, revealing an immune metabolic interaction model with spatial resolution. In the clinical dimension, high levels of GCSH expression are significantly correlated with poor prognosis in patients. In addition, KEGG pathway enrichment analysis [13] [14] and CancerSEA functional annotation confirmed that this factor is deeply involved in metabolic regulation and immune modulation processes. Based on this, we further conducted immune landscape lineage analysis and drug sensitivity prediction, aiming to provide therapeutic strategies for targeting GCSH‐mediated pathological mechanisms.

In summary, this study reveals the immunometabolic role of GCSH in TAMs and emphasizes its promoting effect on tumor progression through MIF‐mediated signal transduction and metabolic adaptation. These findings not only expand our understanding of the biological function of cuproptosis in TME but also establish GCSH as a potential biomarker and therapeutic target, providing new ideas for optimizing antitumor immune response.

## 2. Methods and Materials

### 2.1. Data Acquisition and Processing

The TNBC scRNA seq raw data for this study was sourced from the Gene Expression Omnibus (GEO) database, involving datasets GSE161529 and GSE110686. Among them, the sample composition includes TNBC patient tissues (GSM4909281, GSM4909282, and GSM4909283), healthy control samples (GSM4909253), and TNBC T cell and macrophage‐specific single‐cell datasets extracted by bioinformatics filtering (GSM3011853 and GSM3011854). At the same time, this study obtained the ST dataset GSM6433586 corresponding to TNBC tissue. To further validate clinical relevance, the research team retrieved and extracted gene expression profiles and follow‐up clinical data from 101 cancer patients from The Cancer Genome Atlas (TCGA) database. In addition, in response to the batch effects generated during the integration process of multisource datasets, the research team called the limma [15] and sva [16] R packages in the R language environment to perform data standardization and batch correction. Based on this, the integrated dataset achieved statistical homogenization, ensuring the accuracy of subsequent differential expression analysis (DEA) and functional clustering.

### 2.2. Dimensionality Reduction, Clustering, and Functional Scoring of Single‐Cell Data

This study utilized the Seurat [17] software package to perform dimensionality reduction and clustering analysis on single‐cell transcriptome data. Firstly, import the original UMI counting matrix into the Seurat environment and implement strict quality control: exclude cells with gene detection numbers below 200 or above 2500, and exclude low‐quality cells with mitochondrial gene content exceeding 10%. After quality control filtering, a total of 15,012 high‐quality single cells were retained for subsequent analysis. Subsequently, the LogNormalize method was used to normalize the data (with a scaling factor of 10,000), and the Top 2000 highly variable genes (HVGs) were selected using the FindVariable Features function. Construct a principal component analysis (PCA) model based on the aforementioned feature genes, and select the Top 20 principal components (PCs) as the feature space for subsequent calculations. On this basis, the Louvain algorithm (with a resolution parameter of 0.5) is applied to perform cell clustering analysis. In order to achieve intuitive presentation of high‐dimensional data, this study simultaneously used t‐SNE and UMAP algorithms for nonlinear visualization [18]. In addition, lineage annotation of cell populations was performed based on the expression characteristics of classical marker genes in each cluster [19]. In order to evaluate the enrichment level of specific gene sets at the single‐cell level, the research team integrated UCell, singlescore, and Seurat’s built‐in AddModulus Score method to quantitatively score gene modules. Based on this, statistical tests were conducted to compare the distribution differences of gene module scores among different cell subtypes, in order to analyze the functional preferences of each subgroup.

### 2.3. Functional Identification and Evolutionary Trajectory Analysis of Macrophage Subpopulations

After completing preliminary data screening and preprocessing, the research team conducted in‐depth mining on TAM subpopulations. By using pseudotime trajectory analysis, this study depicts the dynamic state evolution of macrophages in the microenvironment of triple‐negative breast cancer (TNBC). The trajectory heat map generated based on this further reveals significant functional heterogeneity within the population [20]. In addition, to analyze the synergistic effects between cells, the research team used CellCall [21] and CellChat software packages to perform cell communication analysis, in order to infer the ligand receptor interaction network and core pathway strength between different cell lineages [22]. For the assessment of macrophage polarization at the single‐cell level, this study used Seurat’s AddModulus Score function to calculate module scores for M1‐like and M2‐like markers based on authoritative literature and classic markers in the immune gene library. The above module scores reflect the relative enrichment of M1‐ or M2‐related transcription programs in individual cells, rather than exclusive terminal states. The study visualized the spatial differences and distribution characteristics of polarization trends of various macrophage subgroups through t‐SNE embedding and violin plots. It is worth noting that due to the M1/M2 classification being a simplified biological model, it is difficult to fully cover the continuous activation lineage of macrophages in specific spatiotemporal backgrounds.

### 2.4. Construction and Analysis of ST Metabolic Map

This study analyzed the metabolic characteristics of the ST dimension in TNBC tissues. In the data preprocessing stage, mitochondrial genes and specific ribosomal gene sequences were first removed to reduce the noise background. Subsequently, the SCTransform algorithm was used to standardize the sequencing data, and PCA was used to achieve feature dimensionality reduction. Finally, the UMAP algorithm was used to complete the clustering recognition and visualization presentation of cell clusters [23]. In order to intuitively map the gene expression characteristics of tissue in situ, the SpatialEigenPlot function was used to map the spatial features of candidate genes, thereby accurately analyzing the anatomical distribution of specific transcripts in the tumor architecture [24]. In addition, this study utilized the scMetabolism package to conduct metabolic pathway enrichment analysis. By quantitatively scoring the metabolic pathways of different cell subpopulations and displaying their expression abundance in various lineages using DotPlot, the functional preferences of cells were further clarified [24]. On this basis, by constructing a spatial heat map of gene expression, the heterogeneity distribution pattern of metabolism‐related genes in the organizational spatial environment was systematically revealed.

### 2.5. Multidimensional Analysis of Spatial Interaction

This research cooperatively applies RCTD (robust cell type decomposition) and MISTy (multiview intercellular signaling networks) algorithms [25] [26], aiming to fine analyze the spatial interaction landscape inside the TNBC tissue. Firstly, the physical spatial structure of the tissue was established by analyzing the abundance of transcripts and the in situ distribution of cell lineage markers. Subsequently, the RCTD algorithm was used to perform spatial deconvolution, integrating the single‐cell transcriptome reference set with the ST data. Based on this, the study was able to infer the cell type proportions of various cell types in different spatial locations of the organization, achieving cell component analysis at the sublattice level [26]. To further analyze the complex intercellular communication logic, this study introduced the MISTy analysis tool to explore cellular interactions within tissue regions from multiple spatial scales, including intrinsic view, juxta view, and para view. By generating a spatial interaction heat map, the research team successfully identified the spatial interaction patterns between different cell populations under specific anatomical structures [25].

### 2.6. Evaluation of Clinical Prognosis and Analysis of Breast Cancer Cell Function

This study integrated Kaplan–Meier (K‐M) survival analysis, DEA, and functional status correlation analysis to systematically evaluate the prognostic value and biological function of GCSH in tumor cells. The research team calls the survival package in the R language environment to perform survival analysis. In order to scientifically divide the experimental groups, the optimal cutoff value of GCSH expression level was determined using the survminer software package. The samples were divided into high‐expression group and low‐expression group, and the proportion of sample size in each group was ensured to be no less than 30%. Subsequently, a log rank test was performed using the fit function to evaluate the statistical significance of the survival curves between the two groups. At the molecular level, the research team applied the limma [15] package to perform DEA and calculated the log2 fold change (log2FC) of each gene. Based on the GSEA function in the clusterProfiler software package, gene set enrichment analysis was conducted using the Hallmark gene set and KEGG metabolic gene set, respectively, to clarify the core pathways regulated by GCSH [27] [28] [29]. In addition, this study introduced 14 functional states of tumor cells defined in the CancerSEA database [30]. The *Z*‐score parameter [31] in the GSVA (gene set variation analysis) algorithm [32] was used to calculate the comprehensive scores of various functional states, and after standardization by the scale function, the Pearson correlation scatter plot was used to reveal the intrinsic relationship between GCSH expression abundance and tumor malignant functional states such as invasion, cell cycle, and apoptosis.

### 2.7. Immune Landscape Lineage Analysis and Immune Infiltration Assessment

In order to further analyze the shaping effect of GCSH on the tumor immune microenvironment, this study stratified the patient population based on the quartiles of GCSH expression levels. According to the expression abundance from high to low, all samples were divided into four levels: Q1 (top 25% highest expression group) to Q4 (bottom 25% lowest expression group) [33]. Based on this, we systematically evaluated the intrinsic relationship between gene expression and immune modulators, chemokines, and tumor‐infiltrating lymphocytes (TILs) through correlation analysis and statistical inference. On this basis, the research team calculated the average expression scores of immune activation genes, immune suppression genes, chemokines, and human leukocyte antigen (HLA) molecules in each group [33]. Visualize the above data using the pheatmap software package to visually present the fluctuation trend of the immune regulatory network under different GCSH expression abundances. In addition, this study obtained detailed immune infiltration lineage data from the TIMER2.0 public database, aiming to analyze the association between various immune cell subtypes and GCSH expression abundance. The research team quantified the correlation strength between GCSH expression level and TIL spatial proportion by calculating the Spearman correlation coefficient. Furthermore, Fisher’s exact test was applied to verify the independence of GCSH expression abundance and TIL spatial distribution, providing a rigorous mathematical basis for GCSH‐mediated immune remodeling effects.

### 2.8. Characterization of GCSH Mutation Spectrum and Evaluation of Immune Pathway Effects

In this study, the research team systematically collected mutation sites in the GCSH coding region and annotated them at the protein level based on standard transcripts. According to the variation effects of amino acid sequences, mutations are classified as synonymous mutations, missense mutations, and protein truncation mutations (including nonsense mutations and frameshift mutations). By searching authoritative protein functional databases, we focused on annotating the lipoyl binding domain in the GCSH protein sequence. All variant sites were mapped to the full‐length amino acid coordinate system, and their spatial distribution characteristics were characterized through visualization techniques. To evaluate the population frequency attribute of mutations, the research team compared all mutations with the gnomAD database and performed hierarchical annotation based on database inclusion. Using customized scripts, we integrated and displayed the distribution characteristics of mutation types, protein sites, and structural domains. Subsequently, patients were classified into mutant and wild‐type subgroups based on the mutation status of GCSH. Perform DEA using the limma package and calculate the log2FC, based on which the entire genome is sorted. Based on this, utilizing the clusterProfiler software package, GSEA was conducted by combining the Hallmark gene set and KEGG metabolic gene set. After calculating the enrichment score (ES), strict statistical tests and multiple hypothesis correction were performed to visualize the significantly enriched pathways with a threshold of *p* < 0.05. In addition, this study obtained estimates of immune cell infiltration from TCGA cohort using the TIMER2.0 database. We integrated multiple immune deconvolution algorithms to comprehensively evaluate the relative abundance of different immune components and their correlation with GCSH expression. The Wilcoxon rank sum test was applied to compare the statistical differences in immune cell content between high and low gene expression groups, and the immune cell populations with significant differences (*p* < 0.05) were visually presented through a heat map.

### 2.9. Drug Sensitivity Analysis and Prediction of Therapeutic Drugs

This study is aimed at identifying potential candidate compounds that can block GCSH‐mediated oncogenic effects by conducting correlation analysis between GCSH expression abundance and drug response data and performing cMAP (connectivity map) connectivity analysis on differential gene features related to GCSH. Specifically, the research team searched the PRISM drug screening database and systematically calculated the correlation coefficient between GCSH gene expression levels and drug dose–response curves (AUC values) [34–36]. By quantifying this correlation, the study was able to evaluate the predictive power of GCSH expression levels on sensitivity to specific chemotherapy or targeted drugs. In order to further explore targeted strategies with clinical translational potential, we conducted feature‐matching analysis based on the cMAP database [37, 38]. The study first compared the high and low‐expression samples of GCSH in the breast cancer cohort, screened out 150 significantly upregulated and downregulated differential genes, and constructed the GCSH‐associated gene signature. Subsequently, the optimal feature‐matching algorithm XSum (eXtreme Sum) was used to compare the gene features with the transcriptome changes induced by 1288 compounds in the cMAP library and calculate their connectivity score.

### 2.10. Cell Lines

In this study, representative human breast cancer cell lines MCF‐7 (estrogen receptor positive), MDA‐MB‐231 (triple negative), and human normal breast epithelial cell line MCF‐10A as controls were used. For tumor cell lines MCF‐7 and MDA‐MB‐231, the research team used RPMI‐1640 medium containing 10% fetal bovine serum (FBS) and 1% penicillin streptomycin dual antibody for routine culture. For MCF‐10A cells, DMEM/F‐12 medium supplemented with 5% horse serum, 1% bispecific antibody, and 20 ng/mL epidermal growth factor (EGF) was used. All lineage cells were incubated in a constant temperature humidified incubator at 37°C and 5% CO_2_ concentration. In addition, to ensure the stability of experimental results and consistency of phenotype, cells were passaged every 2–3 days, and all in vitro experiments were limited to cell populations with a passage of less than 10 passages.

### 2.11. Quantitative PCR (qPCR)

Total RNA was extracted from cells using an RNA extraction kit, and the RNA concentration was measured using a NanoDrop spectrophotometer. cDNA was synthesized from total RNA using the PrimeScript RT Reagent Kit (Takara, Japan) according to the manufacturer’s instructions. qPCR was conducted using SYBR Green PCR Master Mix (Applied Biosystems, United States) on a CFX96 Touch Real‐Time PCR System (Bio‐Rad, United States). Relative gene expression was calculated using the *ΔΔ*Ct method, with GAPDH as the internal control. All reactions were performed in triplicate, and data were analyzed using the 2^−*ΔΔ*Ct^ method. The primers used in this study were as follows:

GAPDH‐F: 5 ^′^‐CTCCTCCTGTTCGACAGTCAGC‐3 ^′^.

GAPDH‐R: 5 ^′^‐CCCAATACGACCAAATCCGTT‐3 ^′^.

GCSH‐F: 5 ^′^‐GGAAAGACAGAGGAAGGTGGA‐3 ^′^.

GCSH‐R: 5 ^′^‐AGTGGAAACCTGAACGTGGT‐3 ^′^.

### 2.12. Cell Counting Kit‐8 (CCK‐8) Assay

Cell viability was assessed using the CCK‐8 (Dojindo, Japan) assay. MCF‐7 and MDA‐MB‐231 cells were seeded at a density of 2 × 10^3^ cells per well in a 96‐well plate. After incubation for 24, 48, and 72 h, 10 *μ*L of CCK‐8 solution was added to each well, followed by incubation at 37°C for 2 h. The absorbance at 450 nm was measured using a microplate reader. Results were expressed as the percentage of relative cell viability, calculated as the ratio of absorbance compared to the control group.

### 2.13. Cell Colony Formation

The colony formation assay is used to assess the long‐term proliferative capacity of cells. One thousand cells were seeded into a 6‐well plate and cultured for 10 days in complete medium containing 10% FBS (RPMI‐1640 or DMEM). The medium was changed every 3 days during the incubation. After incubation, the colonies were stained with 0.005% crystal violet solution for 30 min at room temperature. The excess dye was removed by washing with PBS, and colonies consisting of more than 50 cells were counted under a microscope. Colony formation efficiency was calculated by dividing the number of colonies per well by the total number of seeded cells, and the results were expressed as the average number of colonies per well. Each experiment was performed in triplicate, and data are expressed as the mean ± standard deviation (SD).

### 2.14. Wound Healing Assay

Cell migration was assessed using the wound healing assay. MCF‐7 and MDA‐MB‐231 cells were seeded at a density of 1 × 10^6^ cells per well in a 6‐well plate. When the cells reached 90% confluence, a wound was created by scraping the cell monolayer with a sterile pipette tip. After wounding, the cells were washed with PBS to remove detached cells and then incubated in complete medium for 24 and 48 h. The wound healing process was observed and imaged using a light microscope. Migration rate was evaluated by measuring the wound width and calculating the percentage of wound closure.

### 2.15. Flow Cytometry Assay for Apoptosis Detection

Apoptosis was assessed by flow cytometry using the Annexin V‐FITC/PI apoptosis detection kit (BD Biosciences, Cat No. 556547), following the manufacturer’s instructions. Briefly, cells were collected after trypsinization, washed twice with PBS, and resuspended in binding buffer. Cells were then incubated with 5 *μ*L of Annexin V‐FITC and 5 *μ*L of propidium iodide (PI) in the dark at room temperature for 15 min. After incubation, 400 *μ*L of binding buffer was added to each sample, and flow cytometric analysis was performed using a BD FACSCanto II flow cytometer (BD Biosciences). Early apoptotic cells were identified as Annexin V‐positive and PI‐negative, while late apoptotic or necrotic cells were identified as Annexin V‐positive and PI‐positive. Data were collected and analyzed using FlowJo software (FlowJo LLC), and the apoptosis rate was calculated as the percentage of Annexin V‐positive cells. Each experiment was performed in triplicate.

### 2.16. Cytokine Assay for TNF‐*α*, IL‐6, and IL‐1*β*

The levels of TNF‐*α*, IL‐6, and IL‐1*β* were measured using enzyme‐linked immunosorbent assay (ELISA) kits (eBioscience, Cat Nos. 88‐7324‐77, 88‐7064‐77, and 88‐7264‐77) according to the manufacturer’s instructions. After treatment, the culture supernatants were collected and centrifuged at 1000 × g for 10 min to remove cell debris. The supernatants were then added to precoated 96‐well plates and incubated with specific capture antibodies. After 2 h of incubation, the wells were washed, detection antibodies were added, and the plates were incubated for an additional 1 h. Subsequently, substrate solution was added, and the reaction was stopped with the stop solution. Absorbance was measured at 450 nm using a microplate reader (BioTek, United States). Cytokine concentrations were determined by comparing the absorbance values to a standard curve, and the results were expressed as fold change relative to the shNC group. All experiments were performed in triplicate.

### 2.17. Animal Experimentation

All animal experiments were conducted in accordance with institutional guidelines and approved by the Animal Ethics Committee. Female NSG mice (6–8 weeks old) were used in the experiment, and they were acclimatized for 1 week before the experiment. Subcutaneous tumor xenograft models were established by injecting 5 million MCF‐7 cells suspended in 100 *μ*L PBS into the right flank of each mouse. Mice were divided into two groups: the shNC group and the shGCSH group, with six mice in each group. Tumor growth was monitored every 3 days by measuring tumor volume with calipers. Tumor volume was calculated using the formula: *V* = (*L* × *W* ^2^)/2, where *L* is the tumor length and *W* is the tumor width. Mice were humanely euthanized using carbon dioxide (CO2) inhalation, with CO2 introduced at a rate of approximately 25% of the chamber volume per minute until respiration ceased, followed by cervical dislocation to ensure death.

### 2.18. Statistical Analysis

All statistical inferences involved in this study were based on the R language (Version 4.4.2) environment. For quantitative data comparison between two groups, based on the normality test results, Student’s *t*‐test or nonparametric Wilcoxon rank sum test was selected. For the evaluation of differences between multiple groups, one‐way ANOVA or Kruskal–Wallis test is used, supplemented by corresponding post hoc analysis to clarify specific differences between groups. At the level of correlation analysis, the Spearman rank correlation coefficient was used to quantitatively evaluate the correlation strength between gene expression abundance, immune cell infiltration level, and functional status score. Based on clinical follow‐up data, this study constructed K‐M survival curves and applied the log rank test to evaluate the significant differences in survival probability between groups. In addition, univariate and multivariate analyses were performed using Cox proportional hazards regression models to identify independent risk factors that affect prognosis. The data visualization process is mainly implemented through R software packages such as ggplot2, pheatmap, and survminer. In order to reduce the risk of false positives caused by multiple comparisons, the study used the Benjamini–Hochberg method to correct for *p* values. Unless otherwise specified, this study sets the false discovery rate (FDR) or adjusted *p* value less than 0.05 as the statistically significant threshold.

## 3. Results

**Figure 1:**
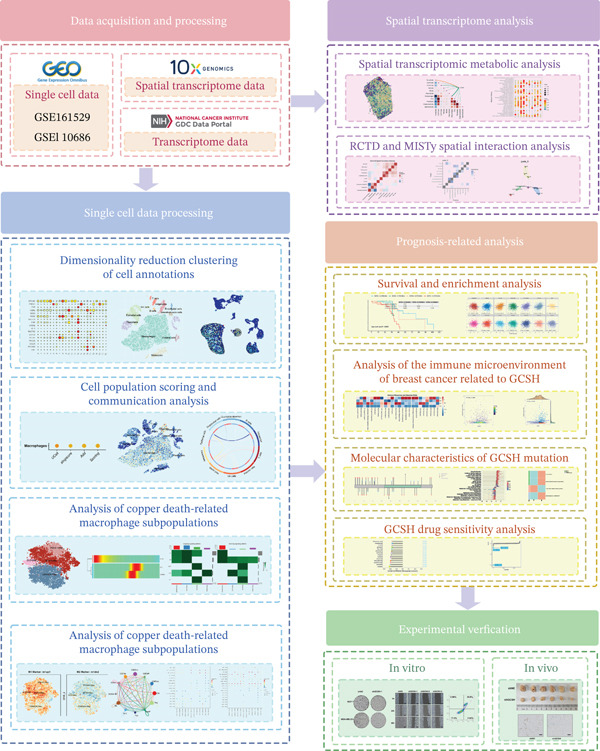
Schematic overview of the study design and analytical workflow.

The overall analytical workflow of this study is illustrated in Figure 1.

### 3.1. Identification of Cell Subpopulations in Tumor Microenvironment and Expression Patterns of GCSH

**Figure 2:**
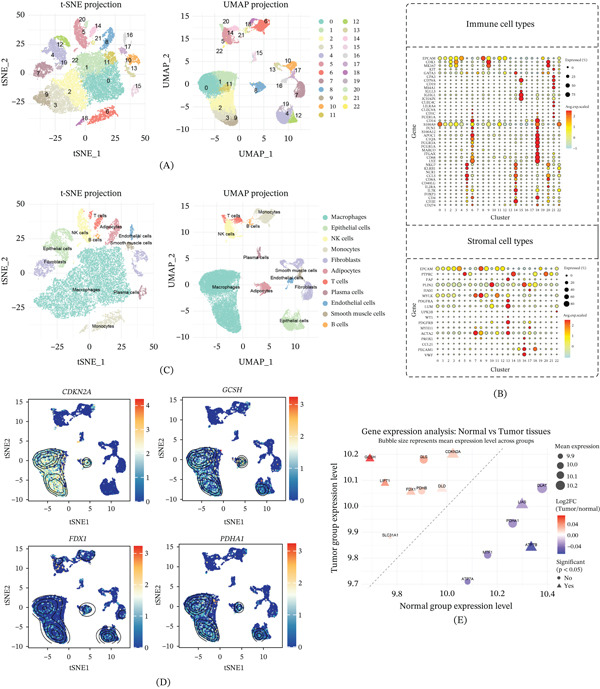
Single‐cell data processing and expression analysis of cuproptosis‐related genes. (A) The t‐SNE and UMAP visualizations of triple‐negative breast cancer single‐cell data are shown. (B) Expression profiles of marker genes across different cell populations. (C) Cell clusters were annotated based on canonical marker genes and visualized using t‐SNE and UMAP plots. (D) UMAP plots showing the expression patterns of cuproptosis‐related genes CDKN2A, GCSH, FDX1, and PDHA1. (E) Bubble plots illustrating the differential expression of cuproptosis‐related genes between normal and tumor tissues, with red indicating upregulation and blue indicating downregulation.

To deeply analyze the cellular heterogeneity within the TME, this study performed unsupervised clustering analysis on single‐cell transcriptome data from tumor tissue and normal tissue adjacent to cancer. Through t‐SNE and UMAP dimensionality reduction projection, we identified 23 cell clusters with unique transcriptional profiles, representing the main immune cell and stromal cell populations (Figure 2A). Based on standard marker genes, cell lineage annotation further clarified multiple immune subgroups, including macrophages, T cells, B cells, DCs, and neutrophils, as well as stromal components such as fibroblasts and endothelial cells (Figure 2B). Among these populations, macrophages constitute the most predominant infiltrating cell type, highlighting their central role in reshaping the TME immune landscape (Figure 2C). It is worth noting that the expression of GCSH is significantly enriched in macrophage clusters, especially in the subsets with high expression of CD68 and APOE (Figure 2D), indicating that GCSH may be closely related to the functional status of TAMs. In contrast, the expression of GCSH is extremely low in lymphocyte and stromal cell populations, indicating significant cell lineage specificity. In addition, compared with normal tissue samples, the expression level of GCSH in tumor samples showed a statistically significant upregulation (Figure 2E). This high‐expression trend is often accompanied by a synchronous increase in the expression of macrophage markers such as CD163 and MRC1. Based on this, it can be inferred that the upregulation of GCSH expression may be potentially associated with the evolution of macrophages towards an immunosuppressive phenotype.

### 3.2. Analysis of Cuproptosis Scoring in Cell Populations and Construction of Intercellular Communication Networks

**Figure 3:**
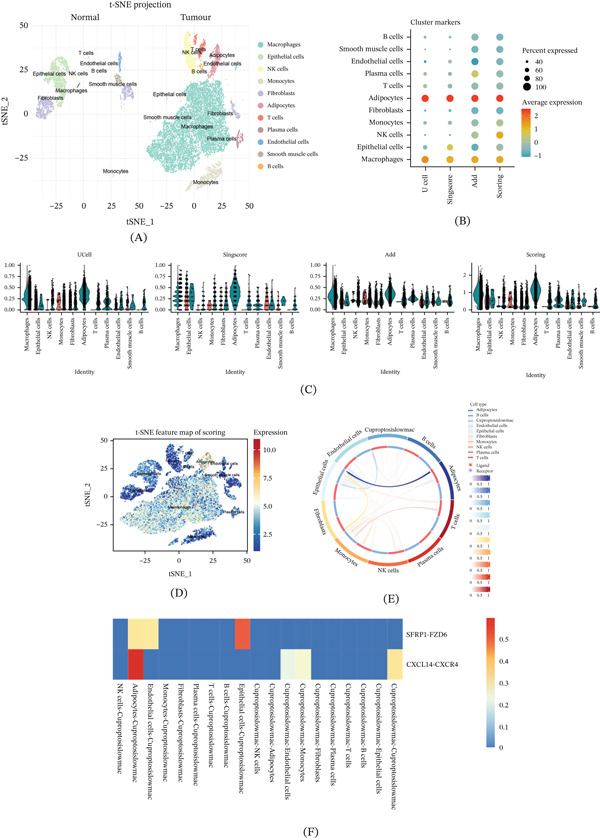
Cuproptosis signature scoring and intercellular communication analysis. (A) The t‐SNE plot compares the distribution of cells between the normal and tumor groups. (B) Heat maps showing UCell, singscore, and AddModuleScore evaluations of cuproptosis‐related gene expression. (C) Violin plots display the detailed expression patterns of UCell, singscore, and AddModuleScore across different cell populations, where each dot represents the average expression level and the percentage of cells expressing the given marker. (D) The t‐SNE projection highlights the spatial distribution of major cell types, with color‐coded scores reflecting the functional activity of the cuproptosis signature. (E) Chord diagram showing communication signals between Cuprotosislowmacrophages and other cell populations. (F) Heat maps depicting SFRP1–FZD6 and CXCL14–CXCR4 communication signals across distinct cell clusters.

To further elucidate the biological properties of macrophages exhibiting high‐level cuproptosis features in the TME, this study performed large‐scale cell population scoring and ligand receptor interaction analysis. The t‐SNE dimensionality reduction projection showed a clear spatial separation trend between immune cells and stromal cell subsets, with macrophages mainly enriched in the tumor area, indicating their deep involvement in the malignant progression process (Figure 3A). The quantitative evaluation of cuproptosis‐related gene expression in each subgroup was conducted by integrating UCell, singlescore, and ADD algorithms. The results showed that the score of the macrophage population was significantly higher than that of other lineages (Figure 3B). The visualization results of the violin chart further confirm this trend (Figure 3C). It is worth noting that high scoring cells exhibit significant heterogeneity within the macrophage population, suggesting the existence of functionally differentiated subsets within this population that may exert distinct biological effects in the microenvironment (Figure 3D). To investigate the potential contribution of low cuproptosis score macrophages in tumor progression, we conducted cell communication analysis and visualized the interaction network using Circos plots. The study observed extensive signal exchange between macrophages and epithelial cells, endothelial cells, and fibroblasts (Figure 3E). Among them, epithelial cells may regulate macrophages through the SFRP1‐FZD6 signaling axis, while adipocytes mainly participate in the interaction through the CXCL14‐CXCR4 axis (Figure 3F).

### 3.3. Revealing Early Activation and Signal Characteristics of GCSH + Macrophage (GCSH + Mac) Through Simulated Temporal Trajectory and Intercellular Communication

**Figure 4:**
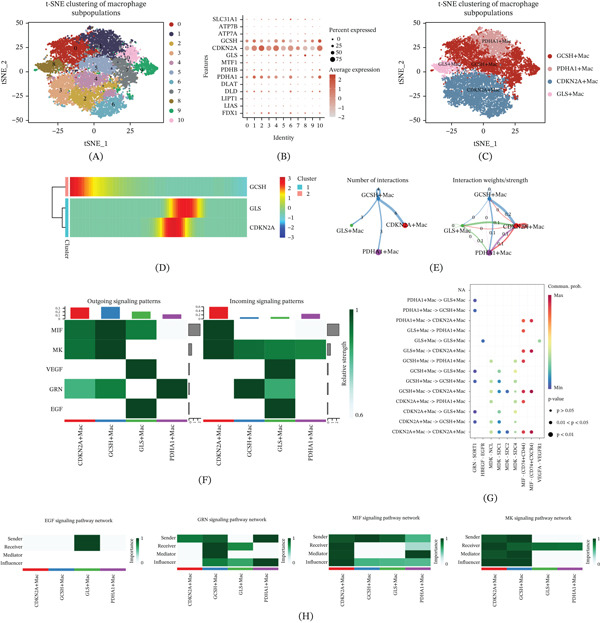
Pseudotime and cell–cell communication analysis of macrophage subpopulations with cuproptosis‐specific characteristics. (A) The t‐SNE clustering plot identifies 11 macrophage subpopulations. (B) Bubble plots display the distribution of cuproptosis‐related genes across different macrophage subclusters. (C) Based on gene expression profiles, four cuproptosis‐specific macrophage subpopulations were merged and visualized using a t‐SNE plot. (D) The pseudotime heat map illustrates the dynamic expression patterns of GCSH, GLS, and CDKN2A during tumor progression. (E) The interaction network shows the number and strength of communication signals between GCSH^+^Mac and the other three macrophage subpopulations. (F) Heat maps depict the input and output signaling intensity of the four subpopulations in the MIF, MK, VEGF, GRN, and EGF signaling pathways. (G) Bubble plots show the detailed ligand–receptor interactions among cell populations, such as MIF–CD74^+^CXCR4. (H) The specific sender, receiver, mediator, and influencer roles of cell subtypes in the EGF, GRN, MIF, and MK signaling pathways are summarized.

To depict the dynamic functions of various macrophage subpopulations associated with cuproptosis during tumor progression, this study performed pseudotime trajectory and intercellular communication analysis. T‐SNE dimensionality reduction projection successfully identified 11 macrophage subclusters with distinct transcriptional features in the tumor microenvironment (Figure 4A). Based on the differential expression patterns of cuproptosis key regulatory factors, we defined four representative functional subgroups: GCSH + Mac, PDHA1 + macrophage (PDHA1 + Mac), CDKN2A + macrophage (CDKN2A + Mac), and GLS + macrophage (GLS + Mac) (Figure 4B,C). The pseudotemporal analysis revealed the spatiotemporal developmental hierarchy among the subgroups mentioned above, with GCSH + Macs significantly enriched at the initial stage of the developmental trajectory (Figure 4D). This discovery suggests that GCSH + Macs may emerge in the early stages of tumor development and potentially participate in the early construction of the procancer microenvironment. In addition, correlation analysis showed a strong correlation between GCSH + Macs and CDKN2A + Macs at the transcriptional level (Figure 4E), indicating that there may be a synergistic functional interaction between the two in the process of tumor evolution. At the cellular communication network level, MIF signaling pathway analysis confirmed that GCSH + Macs exhibited enhanced outward signaling activity (Figure 4F). Specifically, this subgroup actively transmits signals outward through the MIF‐CD74‐CXCR4 interaction axis, suggesting that GCSH + Macs may regulate adjacent immune and stromal cells through paracrine effects, thereby driving tumor progression (Figure 4G). In addition to the MIF axis, the signal pathway interaction map shows differential enrichment of EGF, GRN, and MK pathways in four subtypes of macrophages. Based on this inference, cuproptosis‐associated macrophages adopt diverse communication strategies in reshaping the tumor microenvironment.

### 3.4. Immune Interaction Between GCSH + Macs and T Cell Subsets

**Figure 5:**
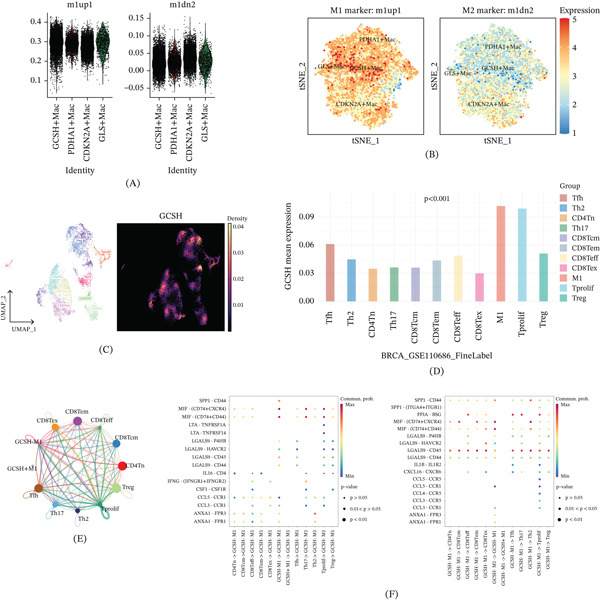
Analysis of macrophage M1 and M2 subpopulations and T cell subsets. (A) Violin plots showing the differential expression of four cuproptosis‐related genes in the m1up1 and m1dn2 clusters. (B) t‐SNE plots depicting the expression patterns of M1 and M2 marker genes across the four cuproptosis‐associated macrophage subpopulations, with red indicating higher expression levels. (B) Spatial distribution maps showing the locations of these 14 clusters within tissue sections. (C) UMAP plots annotating macrophage and T cell subpopulations and displaying GCSH expression within these cell populations. (D) Bar plots quantifying GCSH expression across macrophage and T cell subpopulations. (E) Chord diagrams illustrating cell–cell communication between GCSH^+^M1 or GCSH^-^M1 macrophages and various T cell subsets. (F) Bubble plots representing detailed signaling interactions between specific cell subpopulations.

To further elucidate the immune regulatory function of GCSH + Macs in the tumor microenvironment, this study conducted an in‐depth analysis of the molecular characteristics of this population in macrophage polarization status and T cell subsets. DEA showed that GCSH + Macs were significantly enriched in the M1up1 gene module, while they were significantly downregulated in the M1dn2 module (Figure 5A,B). This suggests that the expression of GCSH is highly correlated with macrophages exhibiting M1‐like proinflammatory transcriptional features. Based on this inference, such transcriptional features reflect the relative enrichment of M1‐related gene expression programs, rather than representing terminal functional polarization states. In addition, expression mapping based on UMAP showed significant distribution of GCSH in proliferating T cells (Tprolif), Th2 cells, and regulatory T cells (Treg) (Figure 5C,D), suggesting that GCSH+‐related macrophages may contribute to the construction of functional heterogeneity in the T cell environment. Cell‐to‐cell communication analysis revealed strong bidirectional interactions between GCSH‐M1 macrophages and multiple T cell subpopulations (Figure 5E). Specifically, the predictive model showed that Th17 cells reverse regulate GCSH‐M1 macrophages through the MIF‐CD74‐C44 and MIF‐CD74‐CR4 signaling axes, indicating that MIF‐mediated interactions may regulate macrophage activation levels. Correspondingly, GCSH‐M1 macrophages transmitted significant output signals to CD8 + effector memory T cells (CD8Tem), exhausted CD8 + T cells (CD8Tex), Th2, and Treg cells through the LGALS9‐CD45 interaction axis (Figure 5F). As this signaling pathway has been proven to mediate T cell suppression and immune tolerance, this further confirms the crucial role of GCSH + Macs in inducing microenvironmental immune suppression.

### 3.5. Spatial Distribution Characteristics and Metabolic Profile of GCSH + Macs

**Figure 6:**
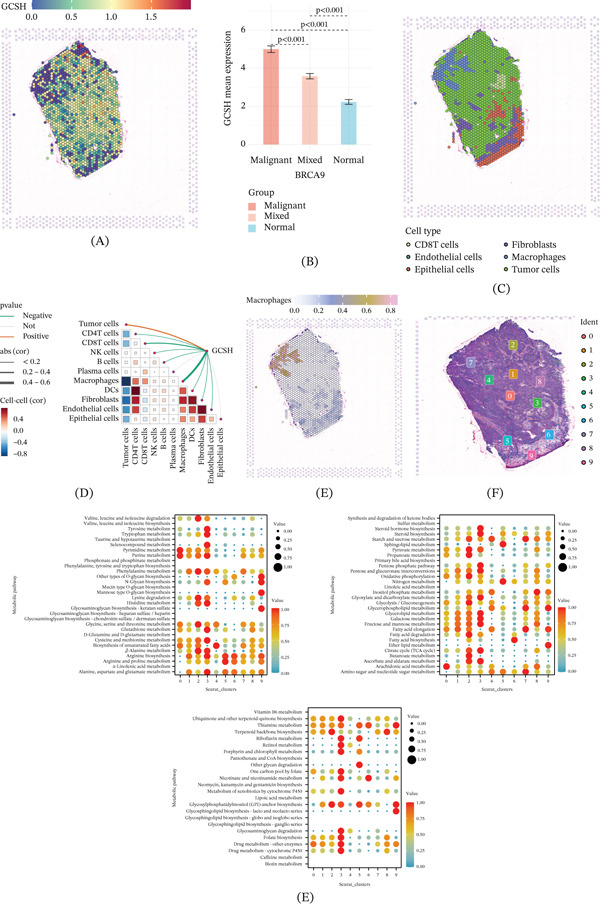
Spatial transcriptomics‐based metabolic analysis. (A) In the spatial transcriptomics maps, each spot represents a microregion of tissue approximately 55 *μ*m in diameter, with deeper red indicating higher GCSH expression. (B) Bar plots show the average GCSH expression levels across different groups, such as malignant or normal regions. (C) Different cell types are represented by distinct colors, illustrating their spatial distribution patterns within the tissue. Red lines indicate positive correlations, green lines indicate negative correlations, and gray lines indicate nonsignificant correlations; the line thickness reflects the magnitude of the correlation coefficient, with thicker lines representing stronger correlations. (D) Within the triangular region, red squares indicate positive correlations, and blue squares indicate negative correlations; the color intensity corresponds to *p* value significance, with darker colors representing more significant *p* values, and the square size is proportional to the absolute value of the correlation coefficient. (E) Macrophage expression positively correlates with color intensity, and (F) the spatial locations of 10 cell populations are displayed on the spatial transcriptomics map. (G) Bubble plots depict metabolic pathway enrichment across the 10 subpopulations, with darker colors indicating greater significance.

In order to further clarify the spatial structure and metabolic properties of GCSH + Macs in TNBC tissues, this study used ST technology to conduct multidimensional analysis. The study first drew a spatial localization map of GCSH expression. The results showed that its expression levels exhibited significant gradient differences in the tumor core, boundary, and adjacent normal tissues: The expression intensity gradually decreased from the tumor core to the peripheral normal areas (Figure 6A,B). This significant spatial expression gradient indicates that the biological activity of GCSH is deeply regulated by the physical niche of the tumor microenvironment. By annotating cell lineages on ST slices, we accurately identified and located key immune and stromal cell populations (Figure 6C). Spatial correlation analysis revealed a significant negative correlation between GCSH‐negative macrophages and tumor cells in spatial distribution (Figure 6D). This suggests that the expression status of GCSH not only determines the polarization tendency of macrophages but may also mediate the physical infiltration and barrier formation of macrophages at the tumor immune junction. Further detailed localization analysis showed that macrophages were codistributed in seven spatial cell clusters (Figure 6E,F). The enrichment analysis of metabolic pathways performed on these clusters showed that phenylalanine metabolism, starch and sucrose metabolism, glycerophospholipid metabolism, fatty acid elongation, and amino sugar and nucleotide sugar metabolism were all significantly activated (Figure 6G). The above findings confirm that GCSH + Macs have an active and unique metabolic phenotype, characterized by significant enhancement of amino acid and lipid metabolism pathways, which may provide metabolic support for their immune suppressive function in the energy‐deficient tumor core area.

### 3.6. Spatial Colocalization and Interactive Landscape of Cuproptosis‐Associated Macrophages

**Figure 7:**
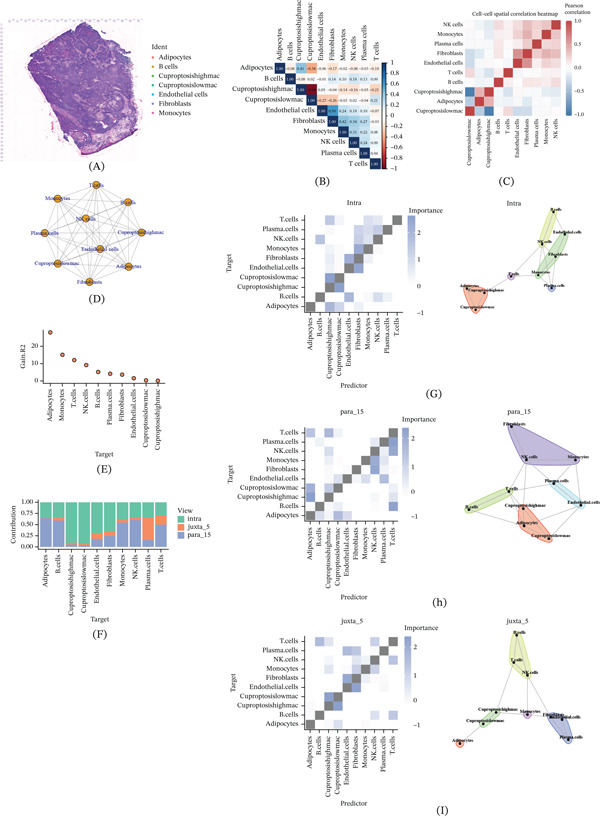
Spatial interaction analysis. (A) Distribution of cell types on tissue sections, with each color representing a distinct cell type. (B, C) Correlation heat maps between different cell types. (D) Interaction network diagrams depicting relationships among various cell types. (E) Single‐view models showing variance among different cell populations. (F) Bar plots comparing the proportions of different cell types under various conditions, such as intra, juxta_5, and para_15. Cell–cell interaction networks and corresponding significance heat maps under (G) intra, (H) para_15, and (I) juxta_5 views, illustrating potential interaction patterns between specific cell types.

To comprehensively depict the spatial interactions between cuproptosis‐associated macrophages and other immune or stromal cell types, this study integrated single‐cell transcriptome and ST data using RCTD and MISTy frameworks (Figure 7A). Spatial correlation analysis showed a significant negative correlation between macrophage clusters highly expressing cuproptosis‐related genes and T cell clusters in spatial distribution (Figure 7B,C). This discovery suggests the possibility of immune compartmentalization in macrophage‐enriched regions or local inhibition of T cell infiltration in this area. The network visualization of intercellular communication further reveals the global connectivity between major cell populations, once again highlighting the core involvement of macrophages in the entire interaction network (Figure 7D). Through the variance decomposition analysis of the MISTy model, it was found that the intraview had the highest variance contribution to the cuproptosis‐related macrophage population. This indicates that local microenvironment signals are the dominant factors shaping the biological behavior of this type of macrophage (Figure 7E,F). Within the scope of the “intrinsic perspective,” macrophages highly expressing cuproptosis genes exhibit extremely strong interaction strength with T cells (Figure 7G), highlighting their potential role in regulating adaptive immune responses. In the para_15 perspective that captures interactions within a moderate spatial range, these macrophages maintain robust communication with T cells and adipocytes (Figure 7H), indicating that these cells may serve as a bridge for immune metabolic cross‐border communication in the ecological niche surrounding the tumor. In contrast, in the juxta_5 perspective representing the short‐range intercellular environment, the interaction intensity between the same macrophage population and neighboring cells is relatively weak (Figure 7I), suggesting the possibility of spatially limited signal transduction activity at tissue junctions.

### 3.7. Prognostic Value and Functional Pathway Enrichment Analysis of GCSH

**Figure 8:**
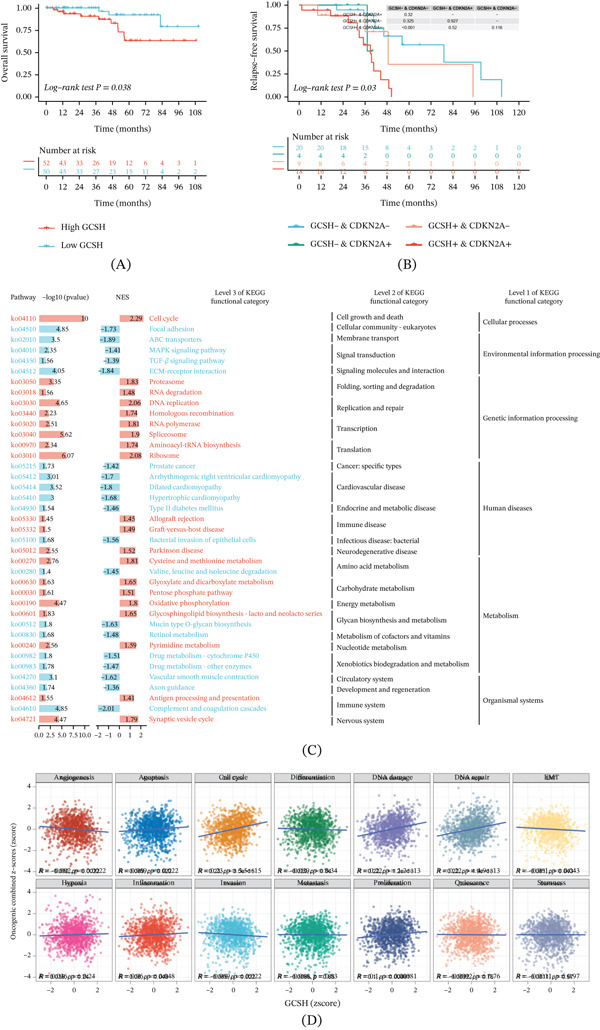
Prognosis‐related survival analysis and KEGG/GSVA enrichment analysis. Kaplan–Meier survival curves evaluating the relationship between GCSH expression levels and overall survival in BRCA patients. The *x*‐axis represents survival time (years), and the *y*‐axis represents the probability of survival beyond a given time point. (A) Red dashed lines indicate the high GCSH expression group, and blue dashed lines indicate the low GCSH expression group. (B) The Kaplan–Meier curves for combined analysis with the CDKN2A gene. (C) Red indicates pathways significantly enriched in the GCSH high‐expression group, whereas blue indicates pathways significantly enriched in the GCSH low‐expression group. (D) Correlation scatter plots depict the relationship between GCSH expression and 14 functional states of tumor cells.

To evaluate the clinical relevance of GCSH in tumor progression, this study conducted survival analysis and pathway mining based on transcriptome data. The K‐M survival curve results show that the overall survival (OS) period of patients in the high‐expression group of GCSH is significantly shorter than that in the low‐expression group (Figure 8A), indicating that this gene is a potential biomarker for poor prognosis of TNBC. Further research has found that coexpression of GCSH and CDKN2A is closely related to a significant reduction in recurrence‐free survival (RFS) (Figure 8B). This indicates that the synergistic activation of these two genes may play a synergistic role in promoting tumor recurrence and invasiveness. To elucidate the molecular mechanism of GCSH driving malignant phenotype, we performed KEGG enrichment analysis on high and low‐expression groups. The results showed a significant enrichment of gene sets related to cell cycle regulation, cell growth, and death pathways (Figure 8C), suggesting that GCSH may play a central role in regulating the balance between cell proliferation and apoptosis. In addition, correlation analysis of the functional status of CancerSEA tumor cells showed that the expression of GCSH was positively correlated with various carcinogenic activities, including apoptosis, cell cycle, DNA damage repair, inflammatory response, and cell proliferation (Figure 8D).

### 3.8. Immunogenomic Characteristic Pedigree of GCSH in TNBC

**Figure 9:**
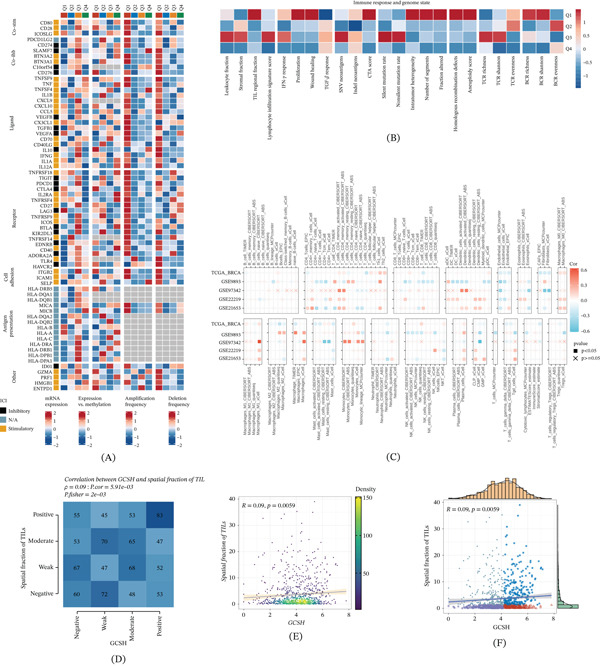
Bulk‐level immune microenvironment analysis. GCSH expression was divided into quartiles (Q1–Q4). In the heat maps (A), from left to right: mRNA expression represents the median of normalized expression levels; expression–methylation shows the correlation between gene expression and DNA methylation *β* values; amplification frequency indicates the difference between the proportion of samples with immune regulator amplification in a specific subtype versus all samples; deletion frequency represents the difference in the proportion of samples with immune regulator deletion in a specific subtype versus all samples. Heat maps in (B) show the within‐group mean scores of each immune response and genomic status for Q1–Q4 subtypes. Rows were standardized so that each score is scaled to the same range. Multialgorithm analysis in SKCM evaluated the Spearman correlation between GCSH expression and various immune‐infiltrating cells, with the color of each square reflecting the correlation coefficient (*p* < 0.05). (C) Red indicates values closer to 1 (positive correlation), blue indicates values closer to −1 (negative correlation), and × marks indicate *p* ≥ 0.05. (E) The Spearman correlation analysis between GCSH expression and the spatial score of tumor‐infiltrating lymphocytes (TILs). (F) Relationship using scatter plots with marginal histograms. (D) The correlation between GCSH expression levels and TIL spatial scores using a contingency table to show the distribution across GCSH expression categories (negative, weak, moderate, and positive) and TIL spatial scores (negative, weak, moderate, and positive).

In order to systematically characterize the immune genome map of GCSH in breast cancer, this study integrated multiple data sets and in‐depth analyzed its immune regulatory molecular expression, somatic copy number variation (SCNA), and epigenetic regulation characteristics (Figure 9A). By comprehensively evaluating the immune epitope presentation score, DNA damage index, and related genomic features, we elucidated the intrinsic relationship between GCSH expression levels and immune infiltration and genomic status. Research has found that patients with GCSH expression levels in the highest quartile (Q1 group) exhibit significant correlations in multiple immune and genomic parameters. Specifically, high expression of GCSH was significantly correlated with the proportion of TIL regions, proliferation score, trauma repair activity, cancer testis antigen (CTA) score, intratumoral heterogeneity, genome fragment number, chromosome variation ratio, homologous recombination defect (HRD), aneuploid score, and abundance of B cell receptor (BCR) with Shannon entropy (Figure 9B). These associations indicate that high expression of GCSH is not only associated with a more active immune microenvironment but also reflects a higher degree of genomic instability. In addition, the analysis of independent breast cancer cohorts revealed a dataset‐specific correlation pattern between GCSH expression and different immune cell populations (Figure 9C), highlighting the background‐dependent role of GCSH in immune regulation. Fisher’s exact test further confirmed a significant positive correlation between GCSH expression levels and TIL spatial distribution scores (Figure 9D,F). This discovery strongly supports the potential of GCSH as a regulatory factor for immune cell infiltration and spatial structural remodeling within the tumor microenvironment.

### 3.9. Molecular Characteristics of GCSH Mutation and Its Association With Immune Microenvironment

**Figure 10:**
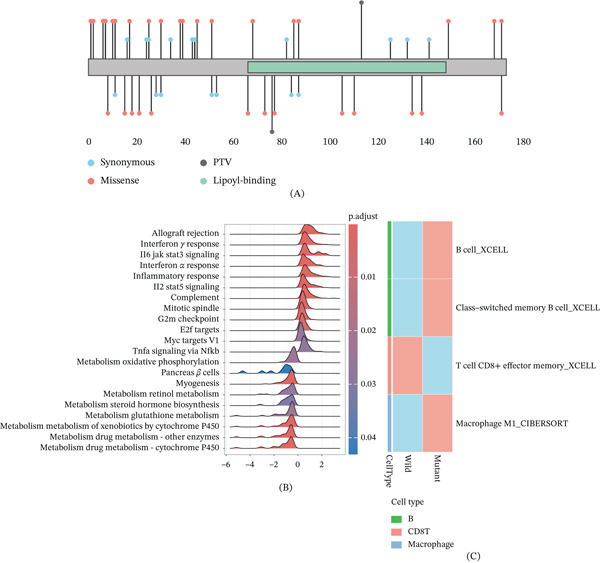
(A) The distribution of coding variations and annotation of functional domains in the GCSH protein coding region is shown, which displays the distribution of coding variations and annotation of functional domains along the full‐length amino acid sequence of the GCSH protein. The horizontal axis represents the position of amino acids, the gray bar represents the main chain of GCSH protein, and the green area indicates the annotated lipoyl binding domain. Different colored dots represent different types of mutations: Blue represents synonymous mutations, red represents missense mutations, and black represents protein truncating variants (PTVs). (B) The pathway enrichment characteristics related to GCSH mutations, with red/blue representing the magnitude of *p* values. (C) The construction of the heat map is based on the average data of immune cell content, with red/blue representing higher/lower average values in the current group.

In order to elucidate the molecular characteristics and potential biological effects of mutations in the coding region of GCSH, we systematically analyzed the distribution of mutation types, pathway enrichment characteristics, and tumor immune cell infiltration patterns. Many types of genetic variation were detected in the coding region of GCSH, mainly missense mutations, accompanied by a certain number of synonymous mutations and a small number of PTVs (Figure 10A). Gene set enrichment analysis based on mutation status showed that multiple immune and inflammation‐related pathways were significantly enriched in GCSH mutation samples, including allograft rejection, interferon gamma response, interferon alpha response, IL6–JAK–STAT3 signaling, IL2–STAT5 signaling, inflammatory response, and TNF‐*α* signaling via NF‐*κ*B, suggesting that GCSH mutation may be closely related to tumor immune activation phenotype (Figure 10B). Further analysis of tumor immune cell infiltration characteristics based on timer2.0 database showed that GCSH mutation status was significantly different from the infiltration level of various immune cell subsets (Figure 10C). Among them, compared with the wild‐type sample, the infiltration level of M1 macrophages in the mutant group was significantly increased, which was consistent with the discovery of the enrichment of immune and inflammatory pathways.

### 3.10. Drug Sensitivity and Potential Therapeutic Targeting of GCSH

**Figure 11:**
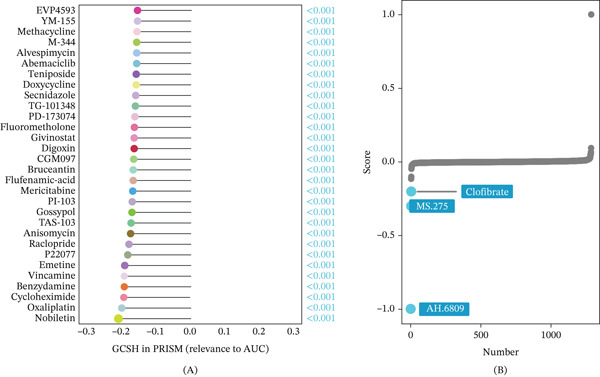
Drug sensitivity analysis. In (A), the *x*‐axis represents the Spearman correlation coefficient between drug IC50 or AUC values and gene expression, and the *y*‐axis shows the Top 30 drugs with the most significant *p* values. Each drug is color‐coded: Red indicates a positive correlation with the gene, and blue indicates a negative correlation. The length of each stick in the lollipop plot reflects the magnitude of the correlation coefficient. In (B), each point represents a compound, with the *y*‐axis showing similarity scores for 1288 compounds obtained by comparing gene‐associated features with cMAP gene signatures using the optimal feature‐matching method XSum (eXtreme Sum).

To evaluate the guiding significance of GCSH in individualized treatment, this study analyzed the association between gene expression and drug sensitivity and screened potential therapeutic drugs. The results of drug sensitivity analysis showed that high expression of GCSH was significantly negatively correlated with the efficacy of various commonly used chemotherapy drugs in clinical practice, including oxaliplatin, pirarubicin, benzamide, and chloramphenicol acetate (Figure 11A). This means that tumors with elevated GCSH levels may exhibit reduced sensitivity or potential resistance to these compounds. Therefore, GCSH‐mediated metabolic reprogramming may play a key role in the chemoresistance mechanism of TNBC. In order to find a solution that can reverse the cancer‐promoting effect of GCSH, this study conducted cMAP connectivity analysis. As a result, the small molecule compound AH.6809 was identified to have the highest reverse matching potential, significantly reversing molecular features associated with GCSH dysregulation, thereby antagonizing the carcinogenic effects driven by GCSH overexpression (Figure 11B). As a prostaglandin receptor antagonist, AH.6809’s prominent performance in cMAP analysis suggests that intervention against GCSH‐related regulatory network or its downstream metabolic pathway may become a new therapeutic strategy to overcome the progress of TNBC and chemotherapy resistance.

### 3.11. Validation of GCSH Function in Breast Cancer Cells by In Vitro Experiments

**Figure 12:**
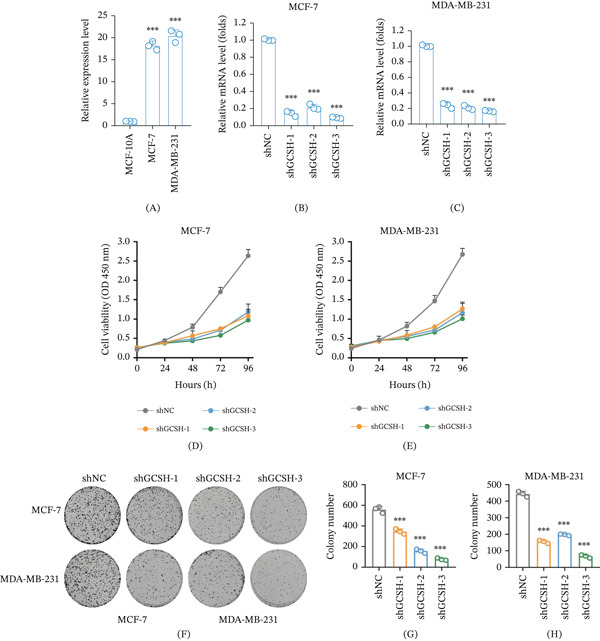
The knockdown effect of GCSH was verified through in vitro experiments. (A) The relative expression of GCSH was measured by quantitative PCR in MCF‐10A, MCF‐7, and MDA‐MB‐231 cells. The relative mRNA levels of GCSH were measured in stable shNC, shGCSH‐1, shGCSH‐2, and shGCSH‐3 transfected (B) MCF‐7 and (C) MDA‐MB‐231 cells. Data are presented as the mean ± SD from three independent experiments. Statistical analysis was performed using *t*‐test, with ∗∗∗ indicating *p* < 0.001. Cell viability was assessed by CCK‐8 assay at different time points (0, 24, 48, 72, and 96 h) in (D) MCF‐7 and (E) MDA‐MB‐231 cells transfected with shNC, shGCSH‐1, shGCSH‐2, and shGCSH‐3. Colony formation was assessed by the colony formation assay in (G) MCF‐7 and (H) MDA‐MB‐231 cells transfected with shNC, shGCSH‐1, shGCSH‐2, and shGCSH‐3. (F) Representative images of the colony formation assays. Data are presented as the mean ± SD from three independent experiments.

In order to verify the reliability of bioinformatics prediction results, this study evaluated the effect of GCSH on breast cancer cell phenotype through a series of in vitro cell function experiments. RT qPCR results showed that compared with the normal breast epithelial cell line MCF‐10A, the expression level of GCSH in breast cancer cell lines (MCF‐7 and MDA MB‐231) was significantly higher (Figure 12A), which was highly consistent with the conclusion that GCSH was highly expressed in tumor tissue in the aforementioned bioinformatics analysis. To investigate the specific function of GCSH, the research team performed gene knockdown in MCF‐7 and MDA‐MB‐231 cells using three shRNA (shGCSH‐1/2/3) sequences targeting GCSH. The qPCR validation results confirmed that compared with the control group (shNC), the GCSH mRNA levels in the knockdown cells of all three groups were significantly reduced (Figure 12B,C), ensuring the effectiveness of subsequent functional experiments. The results of the CCK‐8 proliferation experiment showed that knocking down GCSH significantly inhibited the activity of MCF‐7 and MDA‐MB‐231 cells during continuous monitoring at 0, 24, 48, 72, and 96 h (Figure 12D,E). In addition, the plate cloning experiment further confirmed that silencing GCSH expression significantly reduced the cloning ability of breast cancer cells (Figure 12F,G,H).

### 3.12. GCSH Knockdown Inhibits Migration of Breast Cancer Cells

**Figure 13:**
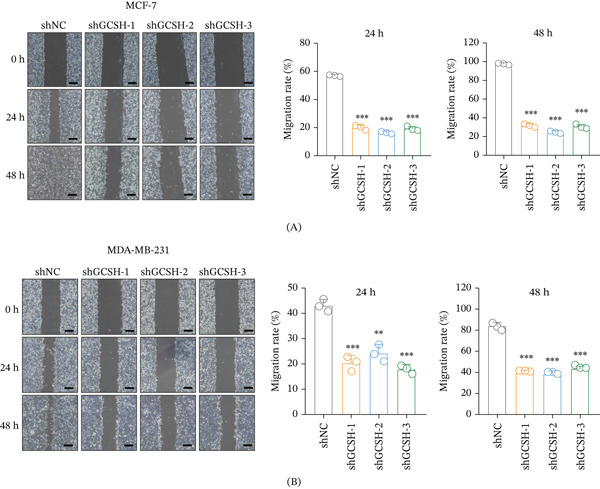
Effect of GCSH knockdown on the migration ability of MCF‐7 cells and MDA‐MB‐231 cells. Wound healing assay was used to assess the migration ability of MCF‐7 cells transfected with shNC, shGCSH‐1, shGCSH‐2, and shGCSH‐3 at 0, 24, and 48 h. (Left) Representative images of wound healing assays. (Right) Quantification of migration rate at 24 and 48 h. Data are presented as the mean ± SD from three independent experiments. Statistical analysis was performed using one‐way ANOVA, followed by Tukey’s post hoc test. *p* < 0.001 indicates significant differences compared to the shNC group. (A) The scale bar = 400 * μ*m. Wound healing assay was used to assess the migration ability of MCF‐7 cells transfected with shNC, shGCSH‐1, shGCSH‐2, and shGCSH‐3 at 0, 24, and 48 h. (Left) Representative images of wound healing assays. (Right) Quantification of migration rate at 24 and 48 h. Data are presented as the mean ± SD from three independent experiments. Statistical analysis was performed using one‐way ANOVA, followed by Tukey’s post hoc test. *p* < 0.001 indicates significant differences compared to the shNC group. (B) The scale bar = 400 * μ*m.

On the basis of confirming that GCSH promotes cell proliferation, this study further evaluated the effect of GCSH on the migration ability of breast cancer cells through the scratch test (wound healing assay). The experimental results showed that during the observation periods of 0, 24, and 48 h, MCF‐7 (Figure 13A) and MDA‐MB‐231 (Figure 13B) cells transfected with shGCSH‐1/2/3 showed a significant decrease in scratch healing rate compared to the control group (shNC). Quantitative analysis showed that knocking down GCSH greatly weakened the lateral migration rate of breast cancer cells.

### 3.13. GCSH Knockdown Promotes Apoptosis and Enhances Inflammatory Cytokine Secretion

**Figure 14:**
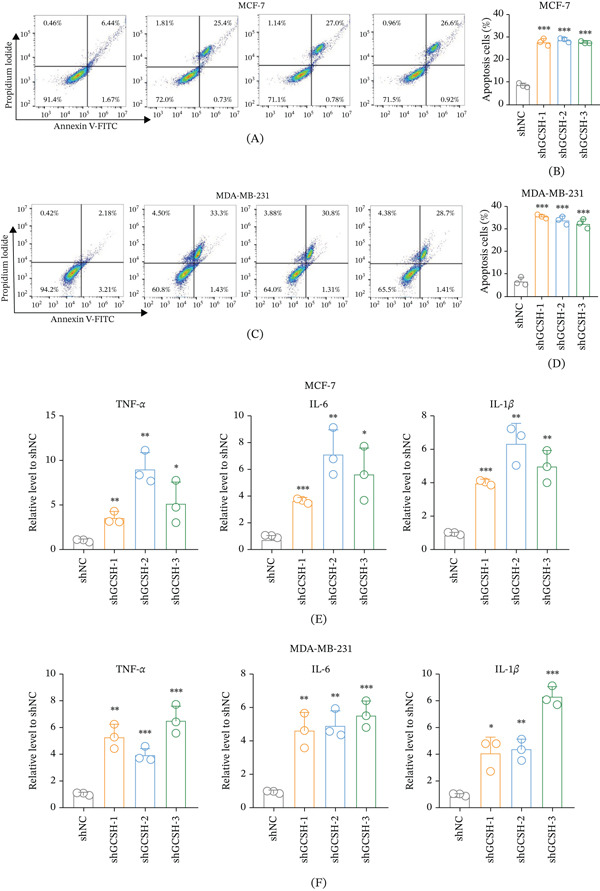
Analysis of the effects of GCSH knockdown in MCF‐7 and MDA‐MB‐231 cells on apoptosis and on macrophage secretion of inflammatory cytokines upon stimulation by these cancer cells. Apoptosis was assessed by flow cytometry using Annexin V‐FITC and propidium iodide staining in (A) MCF‐7 and (C) MDA‐MB‐231 cells transfected with shNC, shGCSH‐1, shGCSH‐2, and shGCSH‐3. (B, D) The quantification of apoptotic cells in MCF‐7 and MDA‐MB‐231 cells, respectively. Data are presented as the mean ± SD from three independent experiments. Statistical analysis was performed using *t*‐test, with ∗∗∗ indicating *p* < 0.001. The relative expression levels of TNF‐*α*, IL‐6, and IL‐1*β* were measured by ELISA in (E) MCF‐7 and (F) MDA‐MB‐231 cells transfected with shNC, shGCSH‐1, shGCSH‐2, and shGCSH‐3. Data are presented as the mean ± SD from three independent experiments. Statistical analysis was performed using *t*‐test, with ∗ indicating *p* < 0.05, ∗∗ indicating *p* < 0.01, and ∗∗∗ indicating *p* < 0.001.

In order to further explore the biological mechanisms by which GCSH maintains the malignant phenotype of tumors, this study evaluated the effects of gene deletions on programmed cell death and immune signaling molecules. Through Annexin V‐FITC/PI dual staining flow cytometry detection, it was found that the proportion of apoptotic cells in the experimental group transfected with shGCSH‐1/2/3 was significantly increased compared to the control group (shNC) in MCF‐7 (Figure 14A,B) and MDA‐MB‐231 (Figure 14C,D) cells. This result shows that GCSH is a key factor for breast cancer cells to escape apoptosis and maintain survival. In addition, ELISA results showed that after knockdown of GCSH, the relative expression levels of proinflammatory cytokines secreted by breast cancer cells, including TNF‐*α*, IL‐6, and IL‐1*β*, were significantly upregulated (Figure 14E,F).

### 3.14. GCSH Knockdown Suppresses Tumor Growth In Vivo

**Figure 15:**
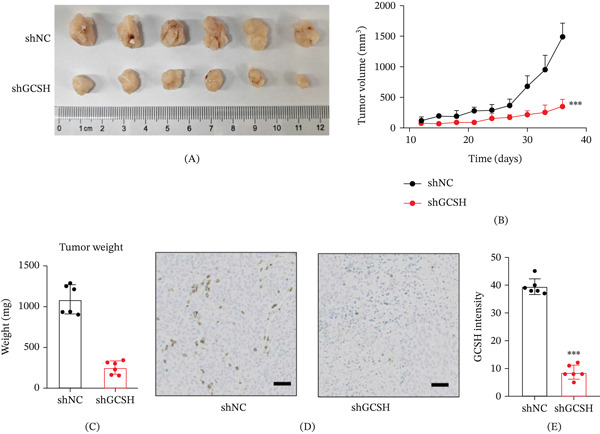
Representative images of tumors from the shNC and shGCSH groups. (A, B) Tumor volume measured at different time points.*n* = 6. (C) Tumor weight recorded at the end of the experiment. Statistical analysis was performed using one‐way ANOVA, followed by Tukey′s post hoc test.  ^∗∗∗^ *p* < 0.001 indicates significant differences compared to the shNC group.

To evaluate the in vivo effect of GCSH knockdown on tumor progression, the research team constructed a nude mouse subcutaneous tumor model (xenogram mouse model). The experimental results showed that compared with individuals injected with control group (shNC) cells, mice injected with shGCSH‐transfected cells exhibited significant inhibition of tumor growth (Figure 15A). Through continuous monitoring of tumor volume, it was found that the growth curve of the shGCSH group was significantly smoother and the growth rate was significantly lower than that of the control group (Figure 15B,C). In addition, immunohistochemical (IHC) staining analysis was performed on the excised tumor tissue. The results confirmed that GCSH protein was strongly expressed in the shNC group, while the staining intensity of the shGCSH group was significantly reduced (Figure 15D). The quantitative evaluation of staining intensity further confirmed that GCSH was effectively silenced at the in vivo level (Figure 15E).

## 4. Discussion

This study systematically analyzed the cell heterogeneity and spatial topology of GCSH expression in the TME of TNBC by using single‐cell sequencing and ST techniques. The data reveals that GCSH is significantly enriched in the macrophage lineage and plays a core regulatory role in reshaping the tumor immune metabolic profile. Based on this, by integrating transcriptional dynamics, intercellular communication networks, spatial distribution characteristics, and clinical correlation analysis, we confirmed that GCSH + Macs constitute a functionally specific subpopulation that closely couples copper‐dependent metabolic cascade reactions with immune regulation and tumor progression processes.

The cell‐type‐specific enrichment of GCSH in macrophages suggests its deep involvement in the biological behavior of TAMs [39]. Compared with normal tissues adjacent to cancer, the abundance of GCSH in tumor specimens was significantly upregulated [40], accompanied by high expression of classic immunosuppressive markers such as CD163 and MRC1 [41] [42]. This phenomenon suggests that the expression of GCSH may be intrinsically associated with the polarization process of macrophages towards a protumor phenotype. In addition, given that macrophages are a key component of TME and profoundly influence malignant processes [43], this macrophage‐dominated expression pattern suggests that GCSH may serve as a key metabolic checkpoint, bridging copper‐induced cell death (i.e., cuproptosis) with macrophage functional reprogramming. Consistent with this logic, cuproptosis score analysis showed that macrophage population scored the highest, further confirming that GCSH + Macs have unique metabolic characteristics and may have higher sensitivity to copper‐dependent oxidative stress pathways that drive tumor immune coevolution.

By using pseudotemporal trajectory analysis to deeply explore the phenotypic heterogeneity of macrophages, the results clarify that GCSH + Macs are located in the early stages of the developmental lineage. This spatiotemporal feature suggests that this subgroup may have recruited at the initial stage of tumor development and played a foundational role in constructing a tumor‐tolerant microenvironment. In addition, this subgroup exhibits a high degree of transcriptional affinity with CDKN2A + Mac, revealing a potential interaction mechanism between the two in coordinating the inflammatory cascade and stress defense programs. It is worth noting that the analysis of the intercellular communication network map identified the MIF‐CD74‐CXCR4 axis [44] [45] as the dominant signaling pathway originating from GCSH + Macs, which has been confirmed to be closely related to macrophage activation and immune escape phenotypes in various solid tumors. Based on this, it is speculated that GCSH + Macs mainly rely on MIF‐mediated paracrine effects to regulate the surrounding immune matrix components, thereby solidifying the tumor‐promoting niche (niche). At the same time, the accompanying enrichment of auxiliary signaling pathways such as EGF [46] and GRN [47] further highlights the multidimensional communication strategies adopted by this subgroup in reshaping the local microenvironment and microecology.

The crosstalk between GCSH macrophages and T cell subsets further elucidates their key properties in immune regulation. Gene module analysis shows that although this subgroup exhibits transcriptional features biased towards M1 activation, it still retains significant phenotypic plasticity, enabling dynamic functional remodeling during tumor progression [48]. It is worth noting that this type of macrophage establishes a tight bidirectional communication network with Treg, Th2 cells, and depleted CD8 ^+^ T cells through MIF‐CD74‐CXCR4 and LGALS9‐CD45 receptor pairs. Specifically, cytokines such as IL‐4, IL‐5, and IL‐13 secreted by Th2 cells can drive macrophages towards M2 polarization, thereby constructing an immunosuppressive microenvironment [49]. Meanwhile, the interaction with Treg and depleted CD8 ^+^ T cells expressing PD‐1/TIM‐3 further weakened the killing activity of effector T cells and induced immune tolerance [50]. The above mechanism suggests that GCSH + Macs may serve as a central node for balancing inflammatory response and immune suppression, maintaining tumor survival by inducing T cell dysfunction [51]. This dual behavior pattern strongly confirms the emerging paradigm that TAMs are not in a discrete M1/M2 binary state but exist in a functional continuum, and it indicates that the metabolic state driven by key factors such as GCSH may be the core element determining the immune regulatory efficacy of macrophages.

The ST analysis provides conclusive evidence that GCSH + Macs exert dual regulation on the immune and metabolic compartments within the TME through specific spatial topological distribution. The data shows that the abundance of GCSH exhibits a significant gradient characteristic of decreasing from the tumor core area to the normal tissue adjacent to the cancer, indicating that its activation has a high degree of microenvironment dependence and is mainly sustained by signals from the tumor source. Further spatial correlation analysis revealed a negative correlation between GCSH‐negative macrophages and tumor cell regions. On the contrary, GCSH + Macs are specifically enriched at the tumor boundary, suggesting their deep involvement in immune infiltration and tissue remodeling processes. At the same time, metabolic pathway enrichment analysis showed that this type of macrophage exhibits high activity in amino acid and lipid metabolism, which is consistent with its metabolic adaptive phenotype supporting tumor growth and immune regulation [52]. In addition, the integration analysis of single‐cell and spatial multimodal data using RCTD and MISTy frameworks further confirmed that macrophages highly expressing cuproptosis‐related genes maintain strong interactions with T cells and adipocytes. This interaction is particularly significant at medium spatial scales, highlighting its key role as a mediator of immune metabolic crosstalk. It is worth noting that the dominant position of intraview variance in the MISTy model further emphasizes that local microenvironment signals are the primary driving factor determining macrophage activation status and spatial function.

From the perspective of clinical translation, the abnormally high expression of GCSH is positively correlated with a significant reduction in OS and RFS of patients, and its coexpression with CDKN2A further deteriorates prognosis. Functional enrichment analysis revealed that GCSH is widely involved in key oncogenic cascade reactions such as cell cycle progression, DNA repair, apoptosis, and inflammatory response, highlighting its multidimensional regulatory role in driving tumor invasiveness and malignant phenotype. In addition, the immunogenomics atlas confirms that high expression of GCSH is closely coupled with high‐intensity immune infiltration, genomic instability, and tumor heterogeneity. This unique molecular feature suggests that GCSH may drive a tumor phenotype of “high immune reactivity but dysfunctional,” where although there is a large recruitment of immune cells within the microenvironment, its effective antitumor activity is inhibited [53]. At the same time, the correlation between GCSH expression abundance and BCR diversity suggests its potential regulatory ability on humoral immune dynamics, thereby further expanding its functional boundaries of immune regulation [54].

At the level of genomic variation, GCSH mutations mainly manifest as missense mutations and are closely related to the immune‐activated tumor microenvironment. The significant enrichment of interferon response, JAK‐STAT, and NF‐*κ*B signaling pathways in mutant tumors suggests that these genetic variations are not simply “passenger mutations” but rather functional changes that may actively reshape inflammation and immune regulatory networks. Consistent with this logic, immune infiltration analysis showed a significant upregulation of M1 macrophages in the mutant group, providing strong evidence for the intrinsic relationship between GCSH mutations and proinflammatory immune microenvironment. Given the central role of macrophage polarization status and interferon signaling in antitumor immunity, it is speculated that GCSH mutations are not only a driving factor for immune microenvironment remodeling but also a key determinant in regulating clinical treatment response.

The pharmacogenomic analysis of this study not only reveals the potential therapeutic significance of GCSH expression dysregulation but also endows relevant findings with deep clinical translational value. Data shows that tumors with high GCSH expression show significant chemoresistance to a variety of chemotherapy drugs, including oxaliplatin, the third‐generation platinum drug commonly used in digestive system malignancies [55] [56] [57], and pirarubicin, the core drug in anthracycline chemotherapy for breast cancer [58]. This phenomenon is highly consistent with the classic hypothesis that metabolic reprogramming confers drug resistance advantages to tumor cells. In addition, through the screening of the cMAP, we identified AH.6809—a nonselective prostaglandin receptor antagonist targeting the blockade of the PGE2‐EP2 axis—as effective in reversing GCSH‐related molecular features [59]. Based on this speculation, pharmacological intervention in the GCSH‐driven signaling network is expected to restore tumor chemotherapy sensitivity and curb its malignant progression. However, it must be cautiously pointed out that the lack of AH.6809 receptor specificity may lead to extensive prostaglandin signal disturbances other than the EP2 pathway, thereby inducing off‐target effects or environment‐dependent pleiotropy reactions. To sum up, these evidences jointly establish that GCSH not only plays an important role as a prognostic biomarker but also is a promising metabolic immune dual target in precision treatment strategies for breast cancer.

We have provided solid mechanistic validation for the aforementioned single‐cell and ST findings through in vivo and in vitro experiments. qPCR data showed that compared with normal epithelial cells, GCSH in breast cancer cell lines showed significantly high abundance expression, suggesting that the upregulation of this molecule has clear tumor endogenous characteristics. Functional experiments further confirmed that silencing GCSH significantly inhibited the proliferation activity, colony‐forming ability, and migration potential of MCF‐7 and MDA‐MB‐231 cells, thereby establishing its core position as a driver of tumor growth and invasion. In addition, flow cytometry and ELISA analysis showed that genetic ablation of GCSH not only induced apoptosis programs but also significantly enhanced the secretion of proinflammatory cytokines such as TNF‐*α*, IL‐6, and IL‐1*β*. This phenotype reveals the dual regulatory function of GCSH in maintaining tumor cell survival while also inhibiting the local inflammatory microenvironment. Consistent with in vitro experimental results, the xenograft model further confirms that the silencing of GCSH significantly suppresses tumor burden at the in vivo level and effectively downregulates tumor protein expression levels. The above experimental evidence strongly confirms the carcinogenic properties of GCSH and highlights its key role in reshaping the endogenous characteristics and immune landscape of tumors. In summary, the organic integration of multiomics data mining and experimental verification has clarified the pivotal position of GCSH as a link between copper‐mediated metabolic regulation, tumor progression, and immune regulation, providing a convincing theoretical basis for its development as a novel therapeutic target.

However, this study still has certain limitations that need to be considered urgently. Firstly, the clinical data included in this study are based on retrospective cohorts, which may to some extent limit the ability to capture the full spectrum of heterogeneity among patients. Secondly, the exact molecular mechanisms by which GCSH regulates macrophage polarization and chemoresistance have not been fully elucidated, and further in‐depth mechanism exploration and prospective treatment studies are needed in the future to fill this knowledge gap.

To sum up, the integrated analysis of this study comprehensively revealed the role of GCSH in the arrangement of immunity, metabolism, and spatial dynamics in the breast cancer microenvironment. GCSH + Macs have been identified as a functionally specific subset of TAMs, located at a critical intersection of cuproptosis regulation, immune communication, and metabolic adaptation. By mediating context‐dependent interactions with T cells and stromal cells, this subgroup strongly drives tumor progression, immune dysfunction, and treatment resistance. Therefore, the “metabolism immune axis” driven by targeting GCSH is expected to become a new intervention strategy to regulate the plasticity of macrophages and enhance antitumor immune response, thus opening up a new treatment path for improving the clinical prognosis of TNBC patients.

## 5. Conclusion

To sum up, this study systematically described the biological characteristic map of GCSH + Macs in TNBC using single‐cell and space transcriptomics techniques. Research has shown that GCSH is significantly enriched in the macrophage lineage and deeply involved in early activation, metabolic reprogramming, and extensive crosstalk with T cells and stromal cells, thereby promoting the construction of an immunosuppressive and protumor microenvironment. Clinical correlation analysis shows that high expression of GCSH is significantly associated with poor prognosis, increased genomic instability, and chemotherapy resistance in patients, highlighting its dual value as a prognostic biomarker and potential therapeutic target. In addition, the functional verification based on breast cancer cell lines and xenotransplantation models further confirmed that GCSH significantly drove the proliferation, migration, and survival of tumor cells. On the contrary, genetic silencing induces cell apoptosis and enhances the secretion of proinflammatory cytokines. Based on the above findings, GCSH has established its core position as a key driver of tumor progression and immune regulation. Therefore, targeted intervention of GCSH‐related signaling pathway is expected to be a novel strategy to regulate macrophage function, curb tumor growth, enhance antitumor immune response, and enhance the therapeutic efficacy of breast cancer.

## Author Contributions

Y.M. and S.W. contributed to the conceptualization of the article. J.G. and T.C. performed bioinformatics analyses, in vitro and in vivo experiments. The original draft was written by J.G. and T.C. S.W. supervised the project, acquired funding, and critically reviewed and finalized the manuscript. J.G. and T.C. have contributed to the work equally and should be regarded as co‐first authors.

## Funding

No funding was received for this manuscript.

## Ethics Statement

All animal experiments were performed in strict accordance with the institutional guidelines for the care and use of laboratory animals and were approved by the Animal Ethics Committee of Huai’an Hospital, affiliated with Xuzhou Medical University (Approval No. HuaiAn‐2024‐0522).

## Conflicts of Interest

The authors declare no conflicts of interest.

## Data Availability

The datasets utilized in our investigation are accessible via the Gene Expression Omnibus (GEO) (https://www.ncbi.nlm.nih.gov/geo/) and The Cancer Genome Atlas Program (TCGA) (https://www.cancer.gov/ccg/research/genome-sequencing/tcga). The unprocessed data can be obtained from jianguoyun at the following link: https://www.jianguoyun.com/p/DeKWAlwQ09brDRj_9Y4GIAA.
